# The gap of water supply—Demand and its driving factors: From water footprint view in Huaihe River Basin

**DOI:** 10.1371/journal.pone.0247604

**Published:** 2021-03-04

**Authors:** Min An, Lijuan Fan, Jin Huang, Wenjing Yang, Hailin Wu, Xiao Wang, Ribesh Khanal

**Affiliations:** 1 Hubei Key Laboratory of Construction and Management in Hydropower Engineering, China Three Gorges University, Yichang, Hubei Province, China; 2 College of Economics & Management, China Three Gorges University, Yichang, Hubei Province, China; 3 College of Hydraulic & Environmental Engineering, China Three Gorges University, Yichang, Hubei Province, China; International Centre for Integrated Mountain Development (ICIMOD), Kathmandu, Nepal, NEPAL

## Abstract

Climate change, population growth, the development of industrialization and urbanization are increasing the demand for water resources, but the water pollution is reducing the limited water supply. In recent years, the gap between water supply and demand which shows water scarcity situation is becoming more serious. Clear knowing this gap and its main driving factors could help us to put forward water protection measures correctly. We take the data of Huaihe River Basin from 2001 to 2016 as an example and use ecological water footprint to describe the demand, with the water carrying capacity representing the supply. We analyze the water supply-demand situation of Huaihe River Basin and its five provinces from footprint view in time and space. Then we apply the Logarithmic Mean Divisia Index model to analyze the driving factors of the ecological water footprint. The results show that: (1) the supply and demand balance of Huaihe River Basin was only achieved in year 2003 and 2005. There is also a large difference between Jiangsu province and other provinces in Huaihe River basin, most years in Jiangsu province per capital ecological footprint of water is more than 1 hm^2^/person except the years of 2003, 2015, and 2016. But other provinces are all less than 1 hm^2^/person. (2) Through the decomposition of water demand drivers, we concluded that economic development is the most important factor, with an annual contribution of more than 60%. Our study provides countermeasures and suggestions for the management and optimal allocation of water resources in Huaihe River Basin, and also provides reference for the formulation of water-saving policies in the world.

## Introduction

Water is the foundation of natural resources and an essential foundation of sustainable social development. However, the total amount of global water resources is very limited. Fresh water resources only account for 2.5% of the total water resources. Of these remaining freshwater resources, about 87% of them were covered with alpine glaciers and permafrost. Freshwater resources that can be used by humanity only account for 0.26% of the total water of the earth [1–5]. With scarcity supply while the growth of population, GDP and the change of consumption structure increasing the water demand with growing at about 1% per year [6,7]. These two sides effects” Limited supply and growing demand” can easily lead to a water crisis. According to the World Water Development Report 2018 estimates that nearly half of the world’s population lives in an area with one month’s water shortage every year, while the population is still increasing year by year, which may reach about 5 billion by 2050 and will cause more serious water shortage.

China is the largest developing country in the world, even its total freshwater resources account for 6% of the global water resources. However, the water consumption per capital is 2200 m3, which is only one fourth of the world level [8]. This make China is one of the 13 countries with the poorest water resources per capital [9,10]. According to China Statistical Yearbook, China’s population has increased from 1.27 billion to 1.39 billion and the regional GDP from 10.9 trillion to 89.6 trillion in the year 2001 to 2018. This rapid increase in the economy and population has brought a massive demand for water resources [11,12]. Compare with the relative slow average growth rate of water supply (0.29%), China is facing huge demand with the average growth rate was 0.54% from 2005 to 2017 [13,14]. If this situation continues, the gap between water supply and demand will become larger and larger. It is predicted that by 2030, China’s total water consumption will reach 700–800 billion m3 per year, while the actual available water resources will be about 800–950 billion m3 and the water demand is close to the limit of available water. China’s water supply and demand imbalance have reached a dire state. Clear knowing the water supply and demand gap has become a top priority for current water resources development [15,16].

From the water supply-demand research type, researches are mainly divided into three categories. The first category is based on statistical data. According to the industrial water and domestic water in Northwest China, the supply and demand of water resources in the basin from the end of 1980s to 2010 were analyzed [17]. The second category is based on an appropriate methodology. For the Tuwei River, through the analysis of multiple feedback and non-linear interactions between the various elements of the system, a complex SD model was established [18]. The third is to use the relationship between supply and demand to study ecological carrying capacity of water, based on the combination model or SWAT model to evaluate the water resources supply and demand [19,20]. From above analysis we found that most of the water situation analysis is from water flows angle. While human activities, economic development and other processes use large amounts of water, but they cannot be reflected in the original form of water resources.

The ecological footprint is a kind of quantitative resource utilization proposed by Canadian ecological economist Wackernagel M [21]. It is an indicator for judging human pressure on the ecosystem and threatening the ecological situation. Kitzes and other researchers expanded the ecological footprint and widely used it in different quantitative evaluation systems and derived the concepts of water footprint, water ecological footprint and so on [22–24]. Water footprint refers to the quantity of water resources required by all products and services consumed by a country (region or person) in a certain period of time. It measures the water consumption of a certain basin by human activities in a certain region [25,26], which nearly cover all the water demand of one region. Therefore, we use ecological water footprint as the demand index of water resources. Water resources carrying capacity refers to the largest scale of water resources system to maintain a virtuous cycle of ecosystem, support its area economic and social development in a specific period of time. It is a "bottleneck" index to measure whether the water resources shortage area can support the coordinated development of population, economy and environment. At some extent this index could reflect the water resources supply of one area [27,28]. Therefore, we use the water ecological carrying capacity represents the supply of water resources. By comparing these two factors, we can judge the security of water ecosystem in this area.

In terms of research object, we choose Huaihe River Basin as a case study. This area has become an important hub connecting the eastern and western regions of China with the implementation of China’s western development strategy. The average annual total water resources of Huaihe River Basin are 79.4 billion m^3^ and the total available water resources is 44.5 billion m^3^. With the water supply from the Yangtze River 5.626 billion m^3^ and from the Yellow River 3.215 billion m^3^, Huaihe River Basin can almost meet the demand of water resources [29,30]. Huaihe River Basin per capita water resources share is about 1/5 of the national per capita water resources, however the average population density of the basin is 4.5 times that of the whole country [31] which make this area a serious water shortage place. Water shortage has brought huge ecological pressure, which has seriously damaged the balance of water in Huaihe River Basin [32]. Obviously, the water supply and demand of the Huaihe River Basin are not balanced [33,34].

For the gap between supply and demand of water resources, water saving is obviously an important measure to alleviate the gap [35,36], but the key to achieve precise water-saving action is to find the key factors that cause the gap. Obviously, compare to the water supply which is not mainly impact by human, the research on the driving factors of water demand is helpful to grasp the influence of different factors on the change of water demand in the past. Accurate identification is conducive to the formulation of water-saving policies and the management of existing water resources.

To calculate the driving factors of water supply- demand gap, Exponential Factorization Method was under our consideration. This Method can be divided into three kinds: Lapsers Index Method, Simple Average Factorization Method and Adaptive Weight Factorization Method. With continuously developed in theory and practice, the model is continuously improved [37,38]. Among them, Log Mean Di’s Decomposition Method is the most widely used model. The LMDI model has the characteristics of not producing residual value and allowing zero in the data, which is clear and objective for the results of influencing factor analysis. Ang., Liu and Ang both think it is the Optimal Decomposition Method. Many scholars use the LMDI to study the energy consumption structure, energy consumption carbon emissions and other issues [39–41]. So, it gives us a lot references to use LMDI Model to explore and analyze the deep-seated problems of water resources in Huaihe River Basin.

Base on above analysis, we choose Huaihe River basin as a case study, with this area’s 2006-2017data, from the perspective of Ecological Footprint of Water (EFW) to analyze the demand of water resources in the Huaihe River Basin, with Ecological Carrying Capacity of Water (ECCW) to measure the supply. Build the relative/absolute indicators of water footprint supply-demand to provide information for environmental protection, water resource planning and management decision basis. Furthermore, the LMDI method is used to analyze the influencing factors of the water supply-demand gap.

To sum up, our paper has the following innovations: (1) describe and analyze the supply and demand of water resources in the Huaihe River Basin from the perspective of water footprint; (2) select EFW and ECCW to describe the demand and supply of water resources.

## Methods and data

### Study area

Huaihe River Basin is located in the east of China, between the Yangtze River and the Yellow River Basin, flowing through Hubei, Henan, Jiangsu, Anhui and Shandong provinces (S1 Fig). The drainage area is about 270000 km^2^, with a population of 178 million. It is one of the seven major water systems in China’s foreign flow area. There are more than 700 main trans-provincial rivers. The number of direct currents ranked first among all water systems in China. There are also many tributaries in Huaihe River System and 21 are primary tributaries with a basin area of more than 1000 km^2^. The geographical location and population area of the Huaihe River Basin are in the forefront of China’s major rivers, so study this important area could give other places some insights.

Huaihe River Basin’s multi-year average per capita water resources are less than 500 m3, which are about 1/5 of the national per capita water resources. While the large use of pesticides in agriculture, the rapid development of high-water consumption and high pollution industries, the rapid growth of population and the concentration of large cities have led to the increase of waste water discharge year by year, which has seriously polluted the water body in the basin. For example, serious water pollution accidents have occurred in the Huaihe River Basin in 2000, 2004, 2007, 2008 and 2010. Among them in 2000, the 6667km^2^ water surface of Shilianghe reservoir was seriously polluted, 200000 people along the bank were faced with drinking water difficulties, and the direct economic loss was more than 10 million. The shortage of water resources and serious water pollution in Huaihe River Basin has seriously restricted the economic development of Huaihe River Basin.

Because of Huaihe River Basin’s important strategic position and severe water supply-demand situation, it becomes a very typical area to study the gap of water supply-demand and its main driving factors from a footprint view.

### Ecological footprint of water demand calculation method (EFW)

Water resources ecological footprint model is established according to the idea of ecological footprint model. Convert water resources consumed by different accounts into corresponding water resources land.

In this article, the status of water resources utilization is subdivided (As Table 1). The calculation formula is as follows:

$$EFw=N\times ef_{w}=r_{w}q_{w}/p_{w}$$ (1)

**Table 1:** Water resources ecological footprint account division table.

Ecological Footprint of Water Resources-Demand Account			
Fishery Account	Aquaculture account		Including fishery production and aquaculture. As the basis of fishery production, water resources provide a very rich source of food for human beings.
Freshwater Account	Agricultural water	Irrigation water	Including water for agriculture, forestry, animal husbandry, water supply to fish ponds and water for rural livestock
		Animal husbandry water	Animal husbandry refers to the production sector that raises livestock and poultry to obtain animal products or livestock by grazing, captive or a combination of the two.
	Industrial water	Industrial water	Mainly including production water, auxiliary production water, and auxiliary production water.
		Construction site water	It mainly includes construction production water, construction machinery water, construction site domestic water, domestic water, and fire protection water.
	drinking water	Urban residents’ consumption of water	Water for residents and public use (including service, catering, freight, post and telecommunications, and construction).
		Consumption water for rural residents	Mainly includes residential water.
Water pollution account	Dilute wastewater to pollute water		Water used to dilute wastewater.
	Dilute acid rain to pollute water		Water used to dilute acid rain.

### Ecological carrying capacity of water supply calculation method (ECCW)

When the utilization rate of water resources exceeds 60%, the ecological environment will be deteriorated [42]. Therefore, when calculating ECCW in this paper, 40% of water resources need to be deducted to maintain basic ecological self-repair and protect the ecological environment.

Calculation formula of ecological capacity of water resources-supply:

$$ES_{w}=N\times es_{w}=r_{w}\psi \{(1-0.6)Q/P_{w}\}$$ (2)

### Absolute indicator of water supply and demand (AIW)

Absolute indicators of water supply and demand can mainly analyze water demand and determine whether water needs to be transferred from the outside. The specific calculation is as follows:

$$Erd=EF_{w}-ES_{w}$$ (3)

### Relative indicators of water supply and demand (RIW)

The relative indicators of water supply and demand can be used to evaluate the intensity of pressure that Huaihe river basin can be withstood and measure the ecological security of water resources in the Huaihe River Basin. The specific calculation is as follows:

$$EFP=EF_{w}/ES_{w}$$ (4)

### Factorization of water ecological footprint based on LMDI method

We divide the water resource use of the Huaihe River Basin into aquatic product consumption, freshwater consumption and wastewater pollution consumption. It is used to characterize the supporting capacity of water resources for human life, economic GDP, society and ecological environment. Therefore, EFW is expressed as follows:

$$EF_{w}=\overset{3}{\underset{i}{\sum }}EF_{wi}=\overset{3}{\underset{i}{\sum }}r_{w}Q_{i}p_{w}$$ (5)

### Driving factors of water footprint in Huaihe River Basin

In order to have a deeper understanding of the ecological footprint of water resources, many scholars have carried out research on the driving factors. Sun [43] focused on the economic driving factors, and made a further elaboration on the water use mode and driving factors in Tibet. There are also relevant studies that show that the impacts of different types of EFW [44], water ecological footprint intensity [45] and population [46] on water ecological footprint cannot be ignored. Therefore, we choose structure, intensity, population and economy as the driving factors of ecological footprint of water resources in our study. The influencing factors of ecological footprint of water resources in Huaihe River Basin were studied by LMDI method. According to Kaya [47–49], the total ecological footprint of water resources can be expressed by the following formula:

$$EF_{wt}=\sum_{i}EF_{wit}=\sum_{i}\frac{EF_{wit}}{EF_{wt}}\times \frac{EF_{wt}}{G_{t}}\times \frac{G_{t}}{N_{t}}\times N_{t}=\sum_{i}S_{it}M_{t}E_{t}N_{t}$$ (6)

In order to carry out this analysis better, it is necessary to consider different factors of water resources ecological footprint change [50,51].

$$\begin{array}{l} \Delta EF_{w_{t}}=EF_{w_{t}}-EF_{w_{t-1}}=\sum_{i}S_{it}M_{t}E_{t}N_{t}-\sum_{i}S_{t-1}M_{t-1}E_{t-1}N_{t}\\ =\Delta EF_{w_{s}}+\Delta EF_{w_{m}}+\Delta EF_{w_{e}}+\Delta EF_{w_{n}} \end{array}$$

$$S_{it}=\frac{EF_{w_{it}}}{EF_{w_{t}}},M_{t}=\frac{EF_{w_{t}}}{G_{t}},E_{t}=\frac{G_{t}}{N_{t}}$$

According to the LMDI method proposed by Ang [52], the impact of each factor on the ecological footprint of water resources can be calculated by the following formula:

$$\Delta EF_{ws}=In\frac{S_{it}}{S_{i(t-1)}}\sum_{i}\frac{EF_{w_{sit}}-EF_{w_{si(t-1)}}}{InEF_{w_{sit}}-InEF_{w_{si(t-1)}}}$$ (7)

$$\Delta EF_{wt}=In\frac{M_{it}}{M_{i(t-1)}}\sum_{i}\frac{EF_{w_{mit}}-EF_{w_{mi(t-1)}}}{InEF_{w_{mit}}-InEF_{w_{mi(t-1)}}}$$ (8)

$$\Delta EF_{we}=In\frac{E_{it}}{E_{i(t-1)}}\sum_{i}\frac{EF_{w_{eit}}-EF_{w_{ei(t-1)}}}{InEF_{w_{eit}}-InEF_{w_{ei(t-1)}}}$$ (9)

$$\Delta EF_{wn}=In\frac{N_{it}}{N_{i(t-1)}}\sum_{i}\frac{EF_{w_{nit}}-EF_{w_{ni(t-1)}}}{InEF_{w_{nit}}-InEF_{w_{ni(t-1)}}}$$ (10)

The above four formulas represent the impact of ecological footprint intensity, economy, population and structure of water resources. The positive value indicates that the index has an increasing effect on the ecological footprint of water resources, and a negative value indicate that the index has an inhibitory effect on the ecological footprint of water resources.

### Formula description

All formula variables in our paper are described as follows (Table 2):

**Table 2:** Formula description table.

variable	Meaning
*EF*_*w*_	the ecological footprint of water resources (hm^3^)
*N*	the total regional population
*ef*_*w*_	the ecological footprint of water resources per capita
*r*_*w*_	the global water resources balance factor
*q*_*w*_	regional consumption total water resources (m^3^)
*p*_*w*_	the world’s average production capacity of water resources (m^3^/hm^3^)
*ES*_*w*_	the carrying capacity of water resources (hm^2^)
*es*_*w*_	the per capita water resources carrying capacity
*r*_*w*_	the water resource balance factor
*ψ*	the average water resource production factor in the Huaihe River Basin, *ψ* = 0.88 [53,54]
*Q*	total water resources (m^3^)
P_*w*_	the average global water production capacity (m^3^/hm^2^)
*Erd*	absolute indicators of water supply and demand
*EFP*	Relative indicators of water supply and demand.
*i*	the type of water resources (that is, the type of consumption of aquatic products, consumption of freshwater resources and consumption of waste-water pollution)
*r*_*w*_	the water resources balance factor
*Q*_*i*_	the type Water consumption (m^3^)
$EF_{w_{i}}$	Total ecological footprint of water in year t
$EF_{w_{it}}$	Ecological footprint of class I water in year t of Huaihe River Basin
*G*_*t*_	Gross national product of Huaihe River Basin in the T year (GDP)
*N*_*t*_	Total population of Huaihe River Basin in year t (person)
$\Delta EF_{w_{s}}$	Changes of water ecological footprint caused by structural factors of water resources ecological footprint
$\Delta EF_{w_{m}}$	Changes of water ecological footprint caused by intensity factors of water resources ecological footprint
$\Delta EF_{w_{e}}$	Changes of water ecological footprint caused by economic factors
$\Delta EF_{w_{n}}$	Ecological footprint of water changes of ecological footprint of water resources caused by population factors
*S*_*it*_	Structure effect of water ecological footprint in Huaihe River Basin
*M*_*t*_	Intensity effect of ecological footprint of water in Huaihe River Basin
*E*_*t*_	Economic effect of ecological footprint of water in Huaihe River Basin
*N*_*t*_	Population effect of ecological footprint of water in Huaihe River Basin

### Data sources

The data on population and GDP are from the statistical yearbook of five provinces from 2001 to 2016 (http://www.stats.gov.cn/tjsj/); the data of water resources are from the Huaihe River Basin Water Resources Bulletin and five provinces’ Water Resources Bulletin from 2001 to 2016 (http://www.hrc.gov.cn/main/szygb/21448.jhtml).

## Results and analysis

### Analysis of water ecological footprint and water carrying capacity

The balance of water supply-demand is an important index to evaluate regional water security. EFW and ECCW are the key indexes to judge the balance of water resources supply- demand from the footprint view. According to the formula (1) and (2), the EFW of Huaihe River Basin in 2001–2016 is calculated and the results are shown in Table 3. The EFW shows an overall upward trend, which means that during this period the demand for water resources in the Huaihe River Basin, continues to rise. We also have the following finds: (1) ECCW per capita increased slightly and then decreased, reaching a peak value of 0.986 hm^2^/person in 2003; other years it is still in a rising trend on the whole from 0.281 hm^2^/person in 2001 to 0.587 in 2016 hm^2^/person which shows that the supply of water is also increasing; (2) ECCW in the year with abundant water resources is significantly greater than that in the year with less total water resources; (3) there is a positive linear relationship between ECCW and the total amount of water.

**Table 3:** Calculation results of the ecological footprint of water in the Huaihe River Basin from 2001 to 2016.

Year	Aquatic product consumption per capita ecological footprint (hm^2^/p)	Ecological Footprint of Freshwater Resources Per Capita (hm^2^/p)	Wastewater pollution per capita ecological footprint (hm^2^/p)	The ecological footprint of water resources per capita (hm^2^/p)	The ecological carrying capacity of water resources per capita (hm^2^/p)	Absolute indicator of water supply and demand (hm^2^/p)	Relative indicators of water supply and demand
2001	0.035	0.375	0.055	0.464	0.281	0.183	1.7
2002	0.037	0.394	0.058	0.488	0.382	0.106	1.3
2003	0.043	0.456	0.067	0.566	0.986	-0.421	0.6
2004	0.043	0.454	0.066	0.562	0.380	0.182	1.5
2005	0.049	0.561	0.076	0.640	0.737	-0.097	0.9
2006	0.049	0.509	0.074	0.631	0.481	0.15	1.3
2007	0.052	0.560	0.082	0.694	0.698	-0.003	1
2008	0.049	0.507	0.072	0.629	0.527	0.102	1.2
2009	0.049	0.499	0.082	0.628	0.414	0.214	1.5
2010	0.046	0.545	0.064	0.656	0.560	0.095	1.2
2011	0.05	0.55	0.074	0.674	0.519	0.155	1.3
2012	0.049	0.512	0.076	0.635	0.434	0.201	1.5
2013	0.049	0.512	0.076	0.656	0.390	0.266	1.7
2014	0.046	0.475	0.066	0.585	0.435	0.149	1.3
2015	0.048	0.463	0.069	0.578	0.497	0.081	1.2
2016	0.046	0.465	0.074	0.582	0.587	-0.006	1

It can be seen from Table 3 that the Huaihe River Basin has been in ecological water footprint deficit for a long time, only ecological water footprint surplus in 2003 and 2005 (See Table 3). This means that the demand for water in the Huaihe River Basin is greater than its supply. At the same time, we find that when the average annual precipitation is rich and the overall water resources are sufficient, the supply-demand of water resources will be balanced. The water consumption has always been high, especially with the increase of GDP. At the same time, the water demand of ecological environment which is used to maintain the water environment ecology and absorb the pollutants from sewage discharge is increasing. In this case, ECCW is too dependent on the precipitation in the basin, which shows that most of the water supply in the Huaihe River Basin comes from the precipitation and the balance of water supply-demand can be achieved in the year with sufficient water while the diversion from the outside should be considered in the dry year.

### Spatial and temporal analysis of ecological footprint of water in five provinces

The Huaihe River basin provides industrial, agricultural and urban water for the cities in the basin. The water environment of the Huaihe River is not only the key factor for the GDP development of the basin, but also the basic support for the development of the five provinces. Therefore, we analyze the five provinces’ EFW and the ECCW which flow through Huaihe River Basin.

From 2001 to 2016, Jiangsu Province has the highest per capita EFW, which is more than 1 hm^2^/person in most years, only in 2003, 2015 and 2016 are less than 1 hm^2^/person and reaching a peak of 1.195 hm^2^/person in 2007. The highest per EFW in Anhui Province is 0.816 hm^2^/person and the lowest is 0.377 hm^2^/person. The overall fluctuation range is relatively large, especially between 2015 and 2016, which is directly reduced from 0.777 hm^2^/person to 0.377 hm^2^/person. This dramatic change may due to the population of Anhui province increasing by more than 30 million in 2016. The fluctuation of EFW in Shandong Province and Henan Province is similar, which is about 0.4 hm^2^/person in most years. For ECCW, Hubei Province and Anhui Province are relative high compare with other provinces. The ECCW per capita in Hubei Province was as high as 0.363 hm^2^/person in 2016 and was as low as 0.151 hm^2^/person in 2001. The difference between the two is 0.212 hm^2^/person and the average carrying capacity of water resources per capita in Hubei is 0.239 hm^2^/person. However, the fluctuation of per capital ECCW in Henan Province, Jiangsu Province and Shandong Province are similar. This further shows that the main factor affecting the ECCW is the total amount of water resources and during study years where with more water resources the ECCW is relatively high.

Combining the difference between the EFW and ECCW, the two indicators’ difference in Jiangsu Province is large, which shows that the water resources supply -demand in Jiangsu Province is large and extremely mismatched. The water resources supply- demand in Henan Province is not much different, which is relatively balanced. Anhui Province, Shandong Province and Hubei Province followed Jiangsu Province in terms of water footprint. But on the whole, the EFW is greater than the ECCW and the demand for water resources is greater than its supply (S2 Fig).

From 2001 to 2016, EFW in the five provinces was greater than ECCW and the absolute index of water supply-demand was less than zero. This means that the regional water resources cannot meet the needs of production and living in that region. Among them, Jiangsu Province has the most serious shortage of supply and the lowest was 0.783 hm^2^ in 2016. The absolute indicator of water supply and demand in Hubei was 0.664 hm^2^; the minimum is 0.403 hm^2^. While the absolute water supply and demand indicators of Henan and Shandong provinces are between 0.221–0.442 hm^2^.

It can be seen from the absolute indicators of water supply and demand are closely related to the number of water resources (as shown in S3 Fig). In those years with abundant regional precipitation and relatively high total water resources, the absolute indicators of water supply and demand are relatively good.

From 2001 to 2016, the relative indicators of water supply and demand in the five provinces were all greater than 1 (S4 Fig). Among them, the largest relative indicator of water supply and demand was in Jiangsu Province. The highest value is 25.77, reaching 6.11 in 2016 and the average relative index of water supply and demand is 13.50.

The relative indicator of water supply and demand in Anhui Province was as high as 2.25 in 2016 and 1.23 in 2013. The average relative indicator of water supply and demand was 1.383 during 2001–2016 in Anhui. The relative index of water supply and demand is greater than 1, which indicates that ECCW in Anhui is less than EFW.

### Calculation results of factor analysis of water ecological footprint

EFW in Huaihe River Basin was decomposed by LMDI and the annual change of EFW influencing factors in Huaihe River Basin from 2001 to 2016 was calculated. To observe the impact of each factor more intuitively, we summarize the four effects. The data of the changes in the ecological footprint of water are converted into their respective contribution rates to the changes in the water ecological footprint (the detail results could be find in Table 4):

**Table 4:** Contribution rate of an ecological footprint effect of various water resources.

Years	S’Contribution rate	M’Contribution rate	E’Contribution rate	N’Contribution rate
2001–2002	29.854	2.346	62.46	5.34
2002–2003	26.453	3.434	64.65	5.463
2003–2004	25.486	1.346	67.432	5.736
2004–2005	30.797	1.689	60.168	7.346
2005–2006	26.7294	2.3446	61.792	9.134
2006–2007	25.1336	3.4614	63.762	7.643
2007–2008	24.052	6.131	61.683	8.134
2008–2009	28.059	1.134	66.673	4.134
2009–2010	16.218	1.346	68.973	13.463
2010–2011	21.344	3.467	66.753	8.436
2011–2012	23.803	4.356	64.678	7.163
2012–2013	25.627	3.465	62.465	8.443
2013–2014	26.832	1.364	62.368	9.436
2014–2015	28.515	1.349	62.673	7.463
2015–2016	24.14	4.643	62.783	8.434

Note: S, M, E, N represent the structural, intensity, economic and Population effects of the ecological footprint of water.

We can intuitively see each factor’s influence to the change of water ecological footprint (S5 Fig). Among them economic development factors have the greatest impact on water supply, with contribution rate of more than 60%. Followed by water use structure, with contribution rate of more than 20%, while intensity effect and population effect have little impact on water supply of Huaihe River Basin, with contribution rate of less than 10%.

For the five provinces, the intensity effect of Hubei Province is greater than the population effect, and the intensity effect of the other four provinces is less than the population effect. The reason may be that the total population of Hubei Province is the smallest among the five provinces, so its GNP is relatively high. Among them, economic effect is the largest contribution, which is the main factors driving the rise of EFW. Since the supply side structural reform, the rapid development of industry and agriculture, the consumption and demand of water resources continue to increase, which promotes the increase of EFW.

## Discussion and suggestions

(1) The balance of supply and demand has been one of the core issues of water resources research, and it is also an important part of the regional water resources evaluation [55]. The purpose of water resources supply-demand balance analysis is to analyze the structure of water resources, solve the contradiction of water and explore new ways of water resources development and utilization [56–58]. EFW can measure the demand of water resources. The greater the demand of social development for water resources, the higher the EFW per capital. ECCW includes social development, resource endowment, supporting status and environmental protection, which comprehensively reflects the supply of water resources. Taking the research results of our paper as an example, during the research period, the EFW in Huaihe River Basin increased from 0.464 hm^2^/person to 0.582 hm^2^/person, showing an upward trend. The ecological footprint of freshwater resources accounted for the largest proportion of EFW, which had a decisive impact on the total per ECCW. However, the water resources carrying capacity increased from 0.281 hm^2^/person to 0.587 hm^2^/person, which was slightly larger than the ecological footprint of water resources. That means the Huaihe River Basin has been in short supply for a long time and there is a large demand for water in agricultural, industrial and domestic water [59]. However, the realization of water resource balance requires certain engineering and policy guarantee measures. So, we suggested that the relevant government departments in each region, based on the current water resources combined with the urban water supply planning, give priority to ensuring the domestic water consumption of residents and coordinate the industrial and other water consumption. In terms of demand, tighter measures can be taken to further reduce water demand and make rational use of existing resources such as rainwater and groundwater to achieve the optimal allocation of water resources to achieve the optimal allocation of water resources [60]. Strengthen the red line management of water resources development and utilization control, and strictly implement the total amount control of water use and water intake. A special agency shall be established to manage water resources in the basin, strengthen the supervision over the operation and maintenance of various engineering measures, ensure the normal and effective operation of various projects and formulate corresponding legal systems, including policies, laws, regulations and regulatory documents.

(2) The driving factors of water resources ecological footprint in Huaihe River Basin are economic effect > structure effect > intensity effect > population effect and the economic effect accounts for more than 60% of the total effect. Industrial structure also has a significant positive driving effect on the increase of water resources ecological footprint. However, water use efficiency and population factors have little influence on the ecological footprint of water resources in Huaihe River Basin, and even show negative influence in some years. Based on the above results, we put forward the following policy suggestions to the water resources management department of Huaihe River Basin.

We should actively explore the internal circulation of economy and optimize the allocation efficiency of water resources. All the provinces in the Huaihe River Basin are big economic provinces in China. The development of scale economy has not yet promoted the decoupling of water resources utilization from economic development. Therefore, the relevant institutions of Huaihe River Basin should take advantage of the development strategy of double circular economy, optimize the allocation of water resources, pay attention to the relationship between water resources utilization efficiency and scale economy continuous and improve the evaluation mechanism of water resources utilization efficiency. Promote the transformation and upgrade industrial structure to improve the high-water consumption nature of the current industrial structure in the Huaihe River Basin. Agriculture and industry are high water consumption industries. Consider about agriculture industries, we also suggest that the proportion of agricultural water in Huaihe River Basin is more than 70% every year, and the proportion of farmland irrigation water is more than 90%. all regions in the Huaihe River Basin should develop the planting scale of agricultural products with low water consumption and high economic benefits reasonably, promote the upgrading of farmland water conservancy facilities and popularize the use of efficient and intelligent water-saving irrigation technology. The key measures to save irrigation water are mainly pipeline water delivery, spray and drip irrigation. In terms of industry, industrial water in Huaihe River Basin is second only to agricultural water, each province in the Huaihe River Basin should restrict the water use of high-water consumption industries, promote industries’ transformation or upgrade water-saving technology. They should also focus on the development of low or no water consumption intensive industries. Deepen the application of water-saving technology and improve the utilization efficiency of water resources. The water-saving policies and regulations, market mechanism and standard system are used to guide peoples’ water-saving production and lifestyle, so as to strictly control the total amount of water consumption in the Huaihe River Basin and improve the utilization efficiency of water resources. Measures to save water mainly include promoting the reuse of recycled water and reclaimed water, and promoting new equipment, new processes and new materials with water-saving, high efficiency, low consumption and low emission.

The rapid population expansion is another factor that affects the increasing demand for water resources. Deal with the relationship among population, resources and environment is the key point of sustainable development. Each region can determine the suitable population range according to the demand and supply of water and the needs for social development, which is the reference basis [61]. In term of water quality, the pollution situation is extremely serious, especially in underdeveloped countries and developing countries. In this regard, we can. We should bring the concept of water conservation into everyone’s mind, popularize water knowledge to increase citizens’ education and publicity.

(3) At present, the world’s resource endowment is extremely unbalanced and the distribution of global water resources is seriously uneven. Nine countries, such as Brazil and Russia, account for more than half of the world’s fresh water resources. Some countries are seriously short of water and the lack of water resources restricts economic development. Especially for developing countries with water shortages, efficient and reasonable using water resources are the current “bottleneck.” The main ways to solve water shortage challenges are open source and saving [62]. In terms of demand, tighter measures can be taken to further reduce water demand and make rational use of existing resources such as rainwater and groundwater.

Climate change will change the spatial pattern of water resources, leading to the change of available water resources. Climate change may affect the change of water demand and water consumption of social and economic development, thus affecting the relationship between water supply and demand of regional economic and social development. At present, the impact of climate change on water resources mainly focuses on seasonal water resources utilization efficiency [63], sustainable development of water resources in arid and semi-arid regions [64], and the water quality of the downstream area [65]. For the Huaihe River Basin, the impact of climate change is uncertain and complex [66]. For example, the northern extreme precipitation is less and more concentrated, which is prone to drought; while the southern extreme precipitation is more and the duration of heavy precipitation is long, which is prone to flood disaster. However, due to the complexity of climate change research, there are great challenges, which need to be further explored by human beings and the progress of research technology. In addition, studies have shown that human activities will reflect climate change and further aggravate the imbalance between supply and demand of water resources in the Huaihe River Basin [30,67]. However, our paper does not focus on the analysis of the impact of climate change on water resources in the Huaihe River Basin, but on the analysis of the existing water resources use and supply, describes the status quo of the balance between supply and demand of water resources in the Huaihe River Basin and excavates the main driving factors. For the future study, we still think its value to further study the impact of climate change on the supply and demand of water resources in the Huaihe River Basin, so as to further analyze its scarcity status and scare coping strategies.

## Conclusion

Although the amount of global water resources is considerable, the differences between water resource supply and demand have become a serious problem not just in China but globally. We used a special study area in China to analyze this water problem from footprint view. The results show that from 2001 to 2016, the Huaihe River Basin was in a state of water supply and demand balance only in 2003 and 2005. In other years, water demand was greater than water supply. This restriction has hindered the sustainable development of economic growth and environment in the river basin. And for the five provinces where the Huaihe River flows, water supply and demand in Jiangsu Province is quite different. Especially in the years when the demand for water resources is large, other measures of water supply may be taken. Water supply and demand in Henan Province is relatively balanced, followed by Anhui Province, Shandong Province and Hubei Province. Facing the gap between water supply and demand, we decompose the influence degree of water resources supply and demand in the Huaihe River Basin by LMDI factors and conclude four effects: economic growth, structural, intensity, and population effect. We found that economic growth is an important factor of water resources supply in the Huaihe River Basin and with proper and reasonable control of economic development could improve the situation of water resources in the Huaihe River Basin. This result provides a new direction for the sustainable development of Huaihe River Basin and also provides a reference for other basins.

## Supporting information

S1 Fig Location of Huaihe River Basin. (TIF)

S2 Fig Water footprint supply and demand map of five provinces. (a) Water footprint supply and demand in Anhui Province; (b) Water footprint supply and demand of Henan Province; (c) Water footprint supply and demand of Shandong Province; (d) Water footprint supply and demand of Hubei Province; (e) Supply and demand of water footprint in Jiangsu Province.
(TIF)

S3 Fig Absolute indicator of water supply and demand in five provinces. (TIF)

S4 Fig Absolute indicator of water supply and demand in five provinces. (TIF)

S5 Fig Effect map of influencing factors in five provinces. (a) Total effect map of five provinces in 2001–2002; (b) Total effect map of five provinces in 2006–2007; (c) Total effect map of five provinces in 2011–2012; (d) Total effect map of five provinces in 2015–2016; (e) Intensity effect of five province; (f) Structure effect of five province; (g) Economic effect of five provinces; (h) Population effect of five provinces.
(TIF)

S1 File (DOCX)

## References

[1] Oelkers E. H., Hering J. G., et al. Zhu C. Water: Is There a Global Crisis? Elements, 2011, 7, 157–162.

[2] Voltz M., Ludwig W., Leduc C., et al.Bouarfa S. Mediterranean land systems under global change: current state and future challenges. Regional Environmental Change, 2018, 18, 619–622.

[3] Wada Y., et al. Bierkens M. F. P. Sustainability of global water use: past reconstruction and future projections. Environmental Research Letters, 2014, 9.

[4] Kanae S. Global Warming and the Water Crisis. Journal of Health Science, 2009, 55, 860–864.

[5] Kim D., Kwon H., Giustolisi O., et al. Savic D. Editorial: Current water challenges require holistic and global solutions. J. Hydroinform., 2018, 20, 533–534.

[6] Dalin C., Konar M., Hanasaki N., Rinaldo A., et al. Rodriguez-Iturbe I. Evolution of the global virtual water trade network. Proceedings of the National Academy of Sciences of the United States of America, 2012, 109, 5989–5994. 10.1073/pnas.1203176109 22474363 PMC3341016

[7] Hoff H. The global water challenge—Modeling green and blue water Preface. Journal of Hydrology, 2010, 384, 175–176.

[8] Yang J., Liu X. J., Ying L. M., Chen X. D., et al. Li M. H. Correlation analysis of environmental treatment, sewage treatment and water supply efficiency in China. Sci. Total Environ., 2020, 708, 8. 10.1016/j.scitotenv.2019.135128 31806337

[9] Jiang Y. China’s water scarcity. Journal of Environmental Management, 2009, 90, 3185–3196. 10.1016/j.jenvman.2009.04.016 19539423

[10] Liu J., et al. Yang W. Water Sustainability for China and Beyond. Science, 2012, 337, 649–650. 10.1126/science.1219471 22879488

[11] Shen D. Post-1980 water policy in China. International Journal of Water Resources Development, 2014, 30, 714–727.

[12] Wang Y., Wan T., et al. Tortajada C. Water Demand Framework and Water Development: The Case of China. Water, 2018, 10.

[13] Wang Y., Zheng C., et al.Ma R. Review: Safe and sustainable groundwater supply in China. Hydrogeology Journal, 2018, 26, 1301–1324.

[14] Yu L., Ding Y., Chen F., Hou J., Liu G., Tang S., et al. An N. Groundwater resources protection and management in China. Water Policy, 2018, 20, 447–460.

[15] Zhu Z., et al. Dou J. Current status of reclaimed water in China: an overview. Journal of Water Reuse and Desalination, 2018, 8, 293–307.

[16] Zuo Q., Liu H., Ma J., et al. Jin R. China calls for human-water harmony. Water Policy, 2016, 18, 255–261.

[17] Liu X., Shen Y., Guo Y., Li S., et al. Guo B. Modeling demand/supply of water resources in the arid region of northwestern China during the late 1980s to 2010. Journal of Geographical Sciences, 2015, 25, 573–591.

[18] Zhu Z., et al. Dou J. Current status of reclaimed water in China: an overview. Journal of Water Reuse and Desalination, 2018, 8, 293–307.

[19] Karamouz M., Mohammadpour P., et al. Mahmoodzadeh D. Assessment of Sustainability in Water Supply-Demand Considering Uncertainties. Water Resour. Manage., 2017, 31, 3761–3778.

[20] Qian L., Wang H., et al. Zhang K. Evaluation Criteria and Model for Risk Between Water Supply and Water Demand and its Application in Beijing. Water Resour. Manage., 2014, 28, 4433–4447.

[21] Girard S., Romary T., Favennec J.-M., Stabat P., et al. Wackernagel H. Sensitivity analysis and dimension reduction of a steam generator model for clogging diagnosis. Reliab. Eng. Syst. Saf., 2013, 113, 143–153.

[22] Yang Y., et al. Cai Z. Ecological security assessment of the Guanzhong Plain urban agglomeration based on an adapted ecological footprint model. Journal of Cleaner Production, 2020, 260.

[23] Zhang P., Deng M., Long A., Deng X., Wang H., Hai Y., et al. Liu Y. Coupling analysis of social-economic water consumption and its effects on the arid environments in Xinjiang of China based on the water and ecological footprints. Journal of Arid Land, 2020, 12, 73–89.

[24] Zhang X., et al. Zhou G. A Scientometric Analysis of Ecological Footprint of Water Resources from 2006–2018. Ekoloji, 2019, 28, 1539–1549.

[25] Fan J. L., Wang J. D., Zhang X., Kong L. S., et al. Song Q. Y. Exploring the changes and driving forces of water footprints in China from 2002 to 2012: A perspective of final demand. Sci. Total Environ., 2019, 650, 1101–1111. 10.1016/j.scitotenv.2018.08.426 30308798

[26] Wang Z., Huang K., Yang S., et al. Yu Y. An input-output approach to evaluate the water footprint and virtual water trade of Beijing, China. Journal of Cleaner Production, 2013, 42, 172–179.

[27] Yang Z., Song J., Cheng D., Xia J., Li Q., et al. Ahamad M. I. Comprehensive evaluation and scenario simulation for the water resources carrying capacity in Xi’an city, China. J. Environ. Manage., 2019, 230, 221–233. 10.1016/j.jenvman.2018.09.085 30290309

[28] Zhu L., Li X., Bai Y., Yi T., et al. Yao L. Evaluation of Water Resources Carrying Capacity and Its Obstruction Factor Analysis: A Case Study of Hubei Province, China. Water, 2019, 11.

[29] Wang X.-J., Zhang J.-Y., Shahid S., Bi S.-H., Elmahdi A., Liao C.-H., et al. Li Y.-D. Forecasting industrial water demand in Huaihe River Basin due to environmental changes. Mitigation and Adaptation Strategies for Global Change, 2018, 23, 469–483.

[30] Wang X.-J., Zhang J.-Y., Shahid S., Xie W., Du C.-Y., Shang X.-C., et al. Zhang X. Modeling domestic water demand in Huaihe River Basin of China under climate change and population dynamics. Environment Development and Sustainability, 2018, 20, 911–924.

[31] Lu Y., Xu H., Wang Y., et al. Yang Y. Evaluation of water environmental carrying capacity of city in Huaihe River Basin based on the AHP method: A case in Huai’an City. Water Resources and Industry, 2017, 18, 71–77.

[32] Fu J., Luo Y., Liu C., et al. Zhu H. Studies on the Environmental Pollution of the Huaihe River, China. Journal of Environmental Protection and Ecology, 2013, 14, 1498–1505.

[33] Zhou L., Sun D., et al. Xu J. Zoning assessment of water environmental supporting capacity for socioeconomic development in the Huaihe River Basin, China. Journal of Geographical Sciences, 2015, 25, 1199–1217.

[34] Kai W., Deyi C., et al. Zhaohui Y. Flood Control and Management for the Transitional Huaihe River in China. Procedia Engineering, 2016, 154, 703–709.

[35] Brentan B. M., Luvizotto E. Jr., Herrera M., Izquierdo J., et al. Perez-Garcia R. Hybrid regression model for near real-time urban water demand forecasting. Journal of Computational and Applied Mathematics, 2017, 309, 532–541.

[36] Hanjra M. A., et al. Qureshi M. E. Global water crisis and future food security in an era of climate change. Food Pol., 2010, 35, 365–377.

[37] Ang B. W. LMDI decomposition approach: A guide for implementation. Energy Policy, 2015, 86, 233–238.

[38] Ma M., Cai W., et al. Cai W. Carbon abatement in China’s commercial building sector: A bottom-up measurement model based on Kaya-LMDI methods. Energy, 2018, 165, 350–368.

[39] Wang W., Zhao D., et al. Kuang Y. Decomposition analysis on influence factors of direct household energy-related carbon emission in Guangdong provinceBased on extended Kaya identity. Environ. Prog. Sustain. Energy, 2016, 35, 298–307.

[40] Song M., Guo X., Wu K., et al. Wang G. Driving effect analysis of energy-consumption carbon emissions in the Yangtze River Delta region. Journal of Cleaner Production, 2015, 103, 620–628.

[41] Liang Y., Niu D., Zhou W., et al. Fan Y. Decomposition Analysis of Carbon Emissions from Energy Consumption in Beijing-Tianjin-Hebei, China: A Weighted-Combination Model Based on Logarithmic Mean Divisia Index and Shapley Value. Sustainability, 2018, 10.

[42] Dai D., Sun M., Xu X., et al. Lei K. Assessment of the water resource carrying capacity based on the ecological footprint: a case study in Zhangjiakou City, North China. Environmental Science and Pollution Research, 2019, 26, 11000–11011. 10.1007/s11356-019-04414-9 30783932

[43] Sun S. A., Bao C., et al. Tang Z. P. Tele-connecting water consumption in Tibet: Patterns and socio-economic driving factors for virtual water trades. Journal of Cleaner Production, 2019, 233, 1250–1258.

[44] Zhao C. F., et al. Chen B. Driving Force Analysis of the Agricultural Water Footprint in China Based on the LMDI Method. Environ. Sci. Technol., 2014, 48, 12723–12731. 10.1021/es503513z 25289879

[45] Jin C., Huang K., Yu Y. J., et al. Zhang Y. Analysis of Influencing Factors of Water Footprint Based on the STIRPAT Model: Evidence from the Beijing Agricultural Sector. Water, 2016, 8, 13.

[46] Zhao C. F., Chen B., Hayat T., Alsaedi A., et al. Ahmad B. Driving force analysis of water footprint change based on extended STIRPAT model: Evidence from the Chinese agricultural sector. Ecol. Indicators, 2014, 47, 43–49.

[47] Gu S., Fu B., Thriveni T., Fujita T., et al. Ahn J. W. Coupled LMDI and system dynamics model for estimating urban CO2 emission mitigation potential in Shanghai, China. Journal of Cleaner Production, 2019, 240.

[48] O’Mahony T. Decomposition of Ireland’s carbon emissions from 1990 to 2010: An extended Kaya identity. Energy Policy, 2013, 59, 573–581.

[49] Zhao C., et al. Chen B. Driving Force Analysis of the Agricultural Water Footprint in China Based on the LMDI Method. Environ. Sci. Technol., 2014, 48, 12723–12731. 10.1021/es503513z 25289879

[50] K. S.. Jung K.H. LMDI Decomposition Analysis for GHG Emissions of Korea’s Manufacturing Industry. Environmental and Resource Economics Review, 2011, 20, 229–254.

[51] Han J. LMDI Decomposition Analysis for Electricity Consumption in Korean Manufacturing. Journal of Energy Engineering, 2015, 24, 137–148.

[52] Liu C.-C. An extended method for key factors in reducing CO2 emissions. Applied Mathematics and Computation, 2007, 189, 440–451.

[53] Lee Y.-J., et al. Peng L.-P. Taiwan’s Ecological Footprint (1994–2011). Sustainability, 2014, 6, 6170–6187.

[54] Su Y., Gao W., Guan D., et al. Su W. Dynamic assessment and forecast of urban water ecological footprint based on exponential smoothing analysis. Journal of Cleaner Production, 2018, 195, 354–364.

[55] Pham T. T., Mai T. D., Pham T. D., Hoang M. T., Nguyen M. K., et al. Pham T. T. Industrial water mass balance as a tool for water management in industrial parks. Water Resources and Industry, 2016, 13, 14–21.

[56] Di C., et al. Yang X. Modeling and Dynamical Analysis of the Water Resources Supply-Demand System: A Case Study in Haihe River Basin. Journal of Applied Mathematics, 2014,.

[57] Donoso G., Melo O., et al. Jordan C. Estimating Water Rights Demand and Supply: Are Non-market Factors Important? Water Resources Management, 2014, 28, 4201–4218.

[58] Asefa T., Adams A., et al. Wanakule N. A Level-of-Service Concept for Planning Future Water Supply Projects under Probabilistic Demand and Supply Framework. Journal of the American Water Resources Association, 2015, 51, 1272–1285.

[59] Li K., Ma T., Wei G., Zhang Y., et al. Feng X. Urban Industrial Water Supply and Demand: System Dynamic Model and Simulation Based on Cobb-Douglas Function. Sustainability, 2019, 11.

[60] Ouda O. K. M. Water demand versus supply in Saudi Arabia: current and future challenges. International Journal of Water Resources Development, 2014, 30, 335–344.

[61] Chai J., Liang T., Lai K. K., Zhang Z. G., et al. Wang S. The future natural gas consumption in China: Based on the LMDI-STIRPAT-PLSR framework and scenario analysis. Energy Policy, 2018, 119, 215–225.

[62] Zou M., Kang S., Niu J., et al. Lu H. A new technique to estimate regional irrigation water demand and driving factor effects using an improved SWAT model with LMDI factor decomposition in an arid basin. Journal of Cleaner Production, 2018, 185, 814–828.

[63] Sun X.-Y., Wang G.-X., Huang M., Hu Z.-Y., et al. Song C.-L. Effect of climate change on seasonal water use efficiency in subalpine Abies fabri. Journal of Mountain Science, 2017, 14, 142–157.

[64] Yu Y., Pi Y., Yu X., Ta Z., Sun L., Disse M., et al. Yu R. Climate change, water resources and sustainable development in the arid and semi-arid lands of Central Asia in the past 30 years. Journal of Arid Land, 2018, 11, 1–14.

[65] Ba W., Du P., Liu T., Bao A., Chen X., Liu J., et al. Qin C. Impacts of climate change and agricultural activities on water quality in the Lower Kaidu River Basin, China. Journal of Geographical Sciences, 2020, 30, 164–176.

[66] Zhang J. Y., Wang G. Q., Pagano T. C., Jin J. L., Liu C. S., He R. M., et al. Liu Y. L. Using Hydrologic Simulation to Explore the Impacts of Climate Change on Runoff in the Huaihe River Basin of China. Journal of Hydrologic Engineering, 2013, 18, 1393–1399.

[67] Zhu Y., Wang W., Liu Y., et al. Wang H. Runoff changes and their potential links with climate variability and anthropogenic activities: a case study in the upper Huaihe River Basin, China. Hydrology Research, 2015, 46, 1019–1036.

