# Supplementary figures and images for: The gap of water supply—Demand and its driving factors: From water footprint view in Huaihe River Basin
Source: PLoS One. 2021 Mar 4;16(3):e0247604. doi: 10.1371/journal.pone.0247604 (PMC7932088; doi:10.1371/journal.pone.0247604)

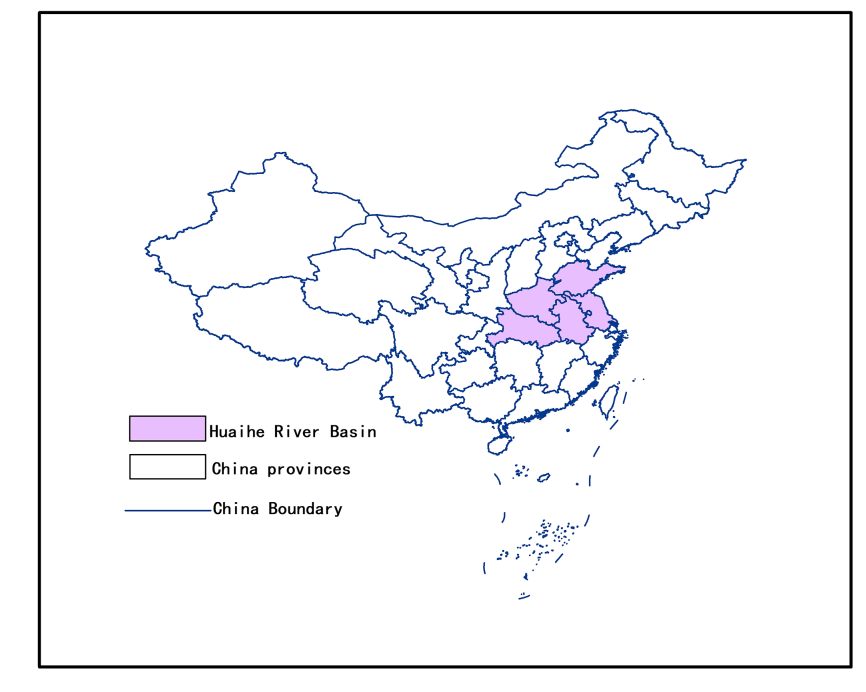

Supplement: S1 Fig — (TIF) [file pone.0247604.s001.tif]

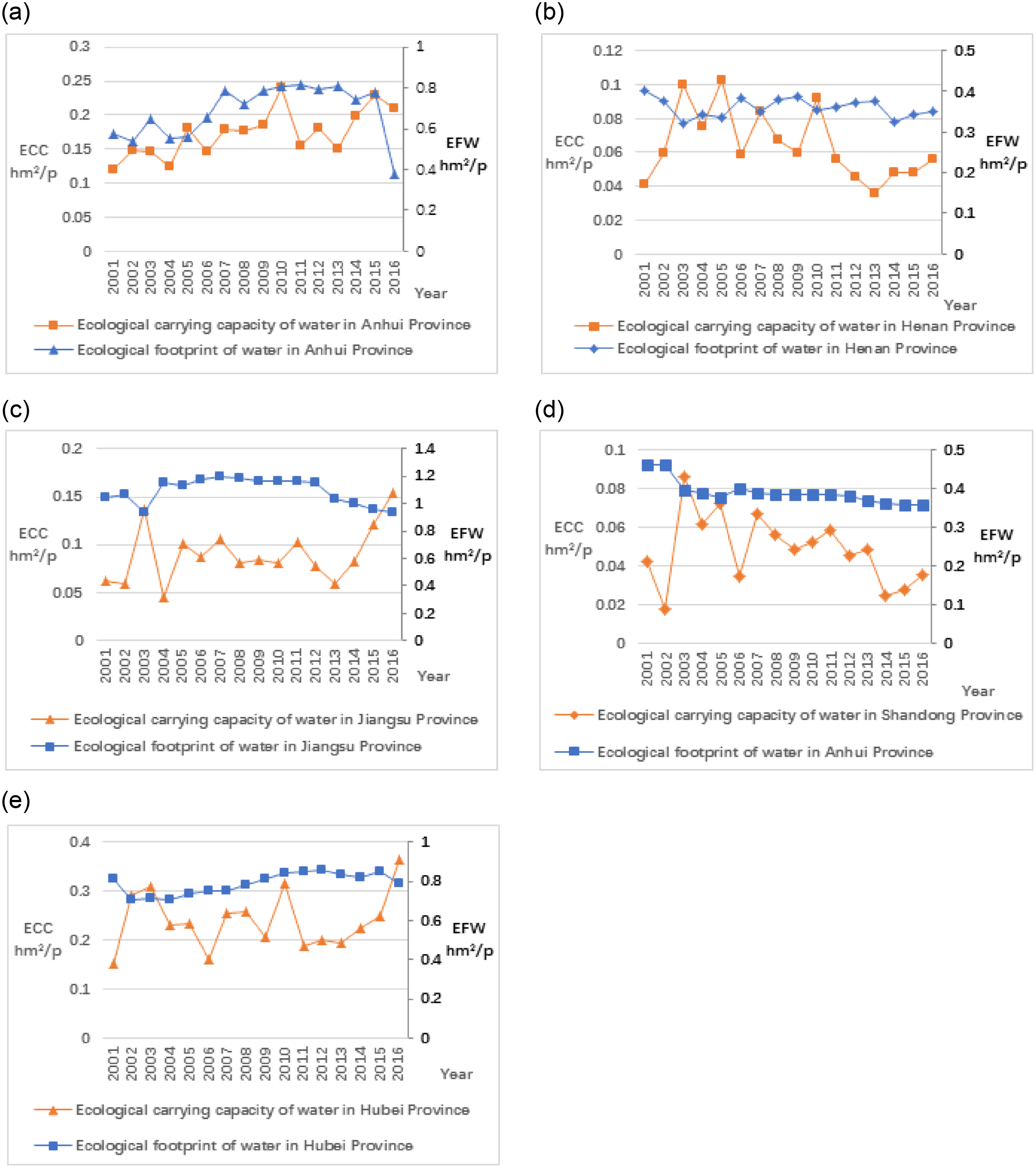

Supplement: S2 Fig — (a) Water footprint supply and demand in Anhui Province; (b) Water footprint supply and demand of Henan Province; (c) Water footprint supply and demand of Shandong Province; (d) Water footprint supply and demand of Hubei Province; (e) Supply and demand of water footprint in Jiangsu Province. (TIF) [file pone.0247604.s002.tif]

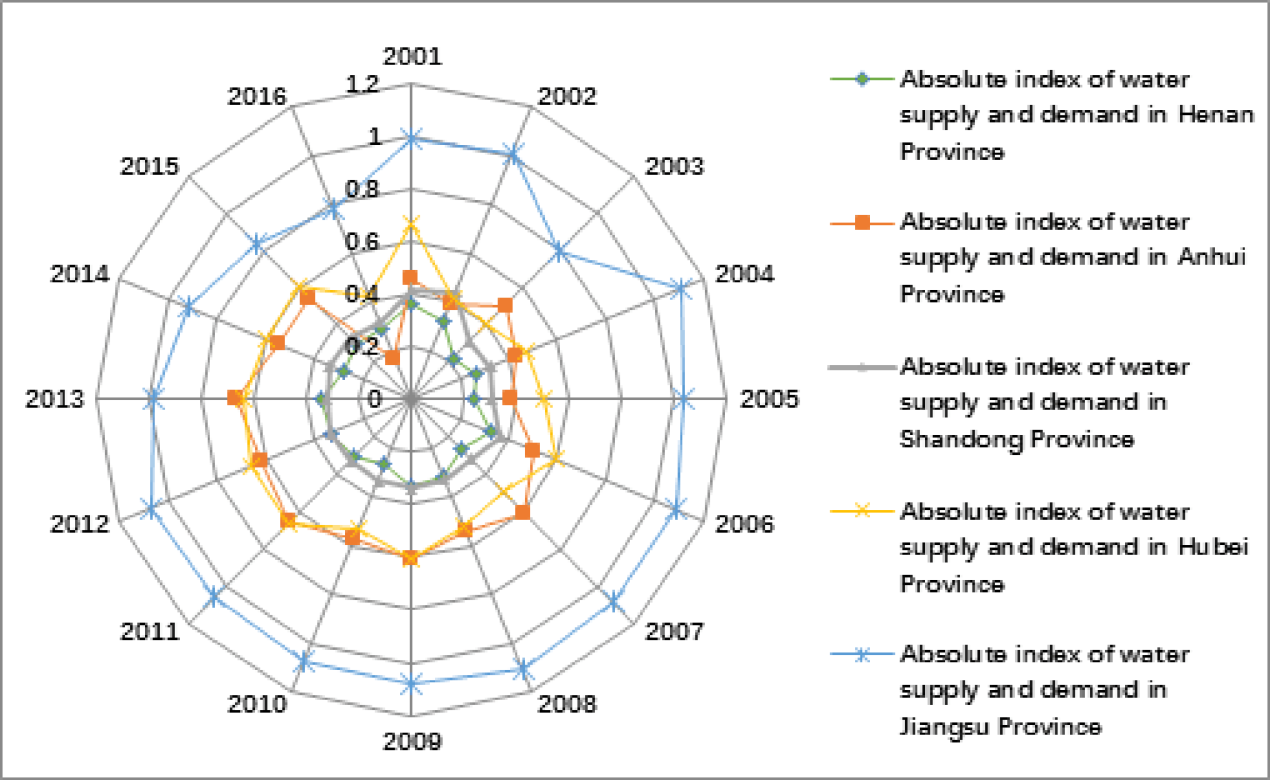

Supplement: S3 Fig — (TIF) [file pone.0247604.s003.tif]

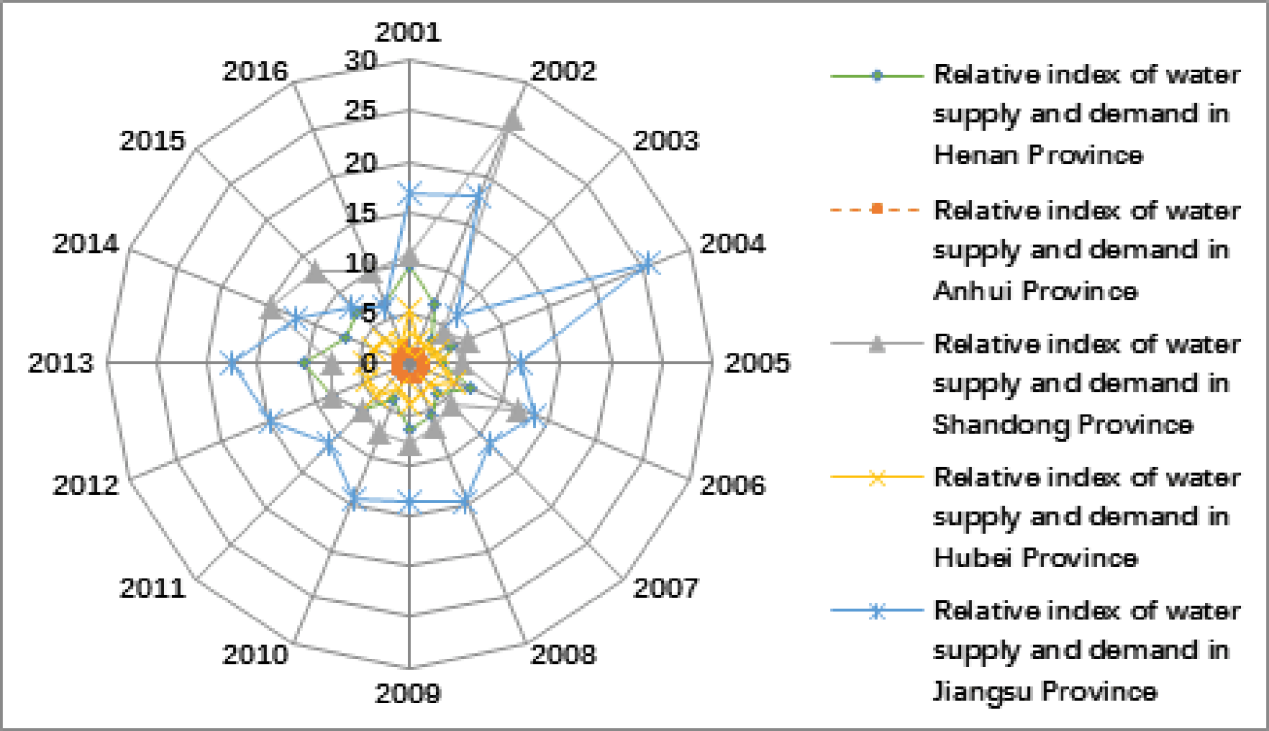

Supplement: S4 Fig — (TIF) [file pone.0247604.s004.tif]

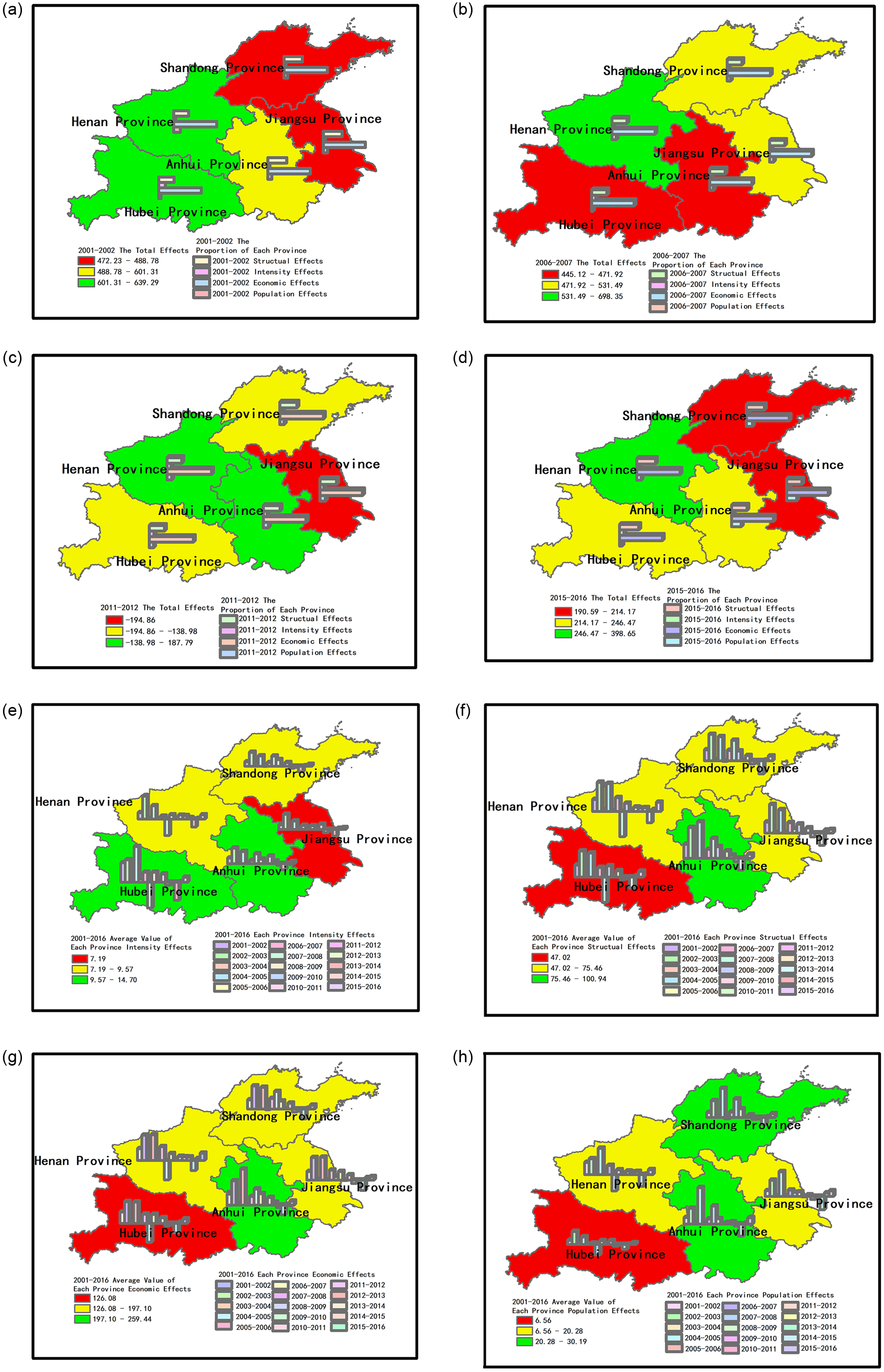

Supplement: S5 Fig — (a) Total effect map of five provinces in 2001–2002; (b) Total effect map of five provinces in 2006–2007; (c) Total effect map of five provinces in 2011–2012; (d) Total effect map of five provinces in 2015–2016; (e) Intensity effect of five province; (f) Structure effect of five province; (g) Economic effect of five provinces; (h) Population effect of five provinces. (TIF) [file pone.0247604.s005.tif]
