# Supplementary material for: The gap of water supply—Demand and its driving factors: From water footprint view in Huaihe River Basin
Source: PLoS One. 2021 Mar 4;16(3):e0247604. doi: 10.1371/journal.pone.0247604 (PMC7932088; doi:10.1371/journal.pone.0247604)
Supplement: S1 File — (DOCX) [file pone.0247604.s006.docx]

**Supporting Information**

**Ecological Footprint of Water Resources in Huaihe River Basin and Five Provinces**

| **Years** | **Huaihe River Basin** | **Henan Province** | **Anhui Province** | **Shandong Province** | **Hubei province** | **Jiangsu Province** |
| --- | --- | --- | --- | --- | --- | --- |
| **2001** | 0.464 | 0.400 | 0.579 | 0.462 | 0.814 | 1.048 |
| **2002** | 0.488 | 0.376 | 0.538 | 0.459 | 0.702 | 1.068 |
| **2003** | 0.566 | 0.321 | 0.649 | 0.397 | 0.713 | 0.934 |
| **2004** | 0.562 | 0.341 | 0.557 | 0.387 | 0.704 | 1.155 |
| **2005** | 0.64 | 0.335 | 0.562 | 0.377 | 0.733 | 1.132 |
| **2006** | 0.631 | 0.382 | 0.654 | 0.401 | 0.751 | 1.180 |
| **2007** | 0.694 | 0.351 | 0.789 | 0.387 | 0.750 | 1.195 |
| **2008** | 0.629 | 0.379 | 0.718 | 0.386 | 0.783 | 1.189 |
| **2009** | 0.628 | 0.388 | 0.787 | 0.384 | 0.813 | 1.162 |
| **2010** | 0.656 | 0.356 | 0.812 | 0.384 | 0.844 | 1.160 |
| **2011** | 0.674 | 0.361 | 0.816 | 0.384 | 0.852 | 1.164 |
| **2012** | 0.635 | 0.374 | 0.797 | 0.376 | 0.856 | 1.152 |
| **2013** | 0.656 | 0.375 | 0.811 | 0.370 | 0.832 | 1.039 |
| **2014** | 0.585 | 0.324 | 0.739 | 0.362 | 0.819 | 0.998 |
| **2015** | 0.578 | 0.344 | 0.777 | 0.357 | 0.851 | 0.955 |
| **2016** | 0.582 | 0.349 | 0.377 | 0.356 | 0.792 | 0.936 |
